# Transillumination of Calot’s Triangle on Laparoscopic Cholecystectomy: A Feasible Approach to Achieve a Critical View of Safety

**DOI:** 10.7759/cureus.9113

**Published:** 2020-07-10

**Authors:** Ramon Vidrio Duarte, Antonio Ramiro Martínez Martínez, Luis H Ortega León, Juan Gutierrez Ochoa, Ariel Ramírez Nava, Gustavo López Sámano, Daniel Torres del Real, Eduardo Vidrio Duarte

**Affiliations:** 1 General Surgery, Hospital General De México "Dr. Eduardo Liceaga", Mexico City, MEX; 2 General Surgery, Hospital General De México “Dr. Eduardo Liceaga”, Mexico City, MEX; 3 Emergency, Hospital Central Sur De Alta Especialidad Pemex, Mexico City, MEX; 4 General Surgery, Hospital Angeles Metropolitano, Mexico City, MEX

**Keywords:** transillumination, laparoscopic cholecistectomy, critical view of safety, calot's triangle, bile duct injury

## Abstract

Background

Laparoscopic cholecystectomy is currently one of the most commonly performed procedures globally. Morbidity of laparoscopic cholecystectomy is low; however, bile duct injury is still a feared complication. Despite worldwide efforts, the global incidence of bile duct injury remains higher for laparoscopic cholecystectomy compared with open cholecystectomy. Despite the general belief that the learning curve and lack of laparoscopic skills represent the most common causes of bile duct injuries, the principal cause is the misidentification of biliary anatomy. The aim of our study is to determine if laparoscopic transillumination is a feasible approach to bile and vascular structures visualization during laparoscopic cholecystectomy because the only other method for real-time visualization is fluorescent cholangiography, which can be cost-prohibitive and requires specialized equipment and training.

Materials and methods

We performed a retrospective comparison of outcomes between the transillumination approach in 10 patients receiving laparoscopic cholecystectomy (group A) and a control group of 50 conventional laparoscopic cholecystectomy patients (group B). We compared demographic data, type of surgery, operative time, bleeding, intraoperative and postoperative complications, and hospital stay. We used a conventional four-port positioning for laparoscopic cholecystectomy, and a 5-mm/30° scope was used as a light source and placed behind the area identified as Calot’s triangle.

Results

Group A consisted of 10 patients (9 women, 1 man), with a mean age of 50.7 (± 17.4) years. The mean body mass index (BMI) in group A was 26.8 (± 0.65) kg/m^2^. In group A, three of the cholecystectomies were conducted as emergency procedures. Group B consisted of 50 patients (40 women, 10 men), with a mean age of 49.7 (±15.2) years. The mean BMI in group B was 27.5 (±4.5) kg/m^2^, and two cholecystectomies were emergency procedures. In comparing the transillumination approach with conventional cholecystectomy, we found no statistical differences in operative time, bleeding, complications, or mean hospital stay.

Conclusions

Laparoscopic transillumination is a feasible method for real-time visualization of Calot’s triangle structures. Our initial experience with transillumination did not provide better outcomes than conventional cholecystectomy.

## Introduction

Laparoscopic cholecystectomy (LC) is currently one of the more common surgical procedures worldwide. In 2006, in the United States, approximately 917,000 LCs were performed [1]. LC is a safe procedure with low complication rates, especially when it is performed electively. Nevertheless, bile duct injury (BDI) remains a serious complication that concerns both the patient and the surgeon due to its impact on quality of life, survival, and medicolegal issues. The incidence of BDI is in LC 0.32% to 0.52%, which is high compared to the 0.1% to 0.2% rate reported for open cholecystectomy [1]. However, the incidence of BDI has declined; from 1994 to 1999, the pooled incidence was 0.52% to 0.84%, and from 2010 to 2014, the incidence was 0.02% to 0.4% [1]. The incidence is higher in developing countries [1,2].

Many techniques have been proposed to obtain intraoperative imaging of the bile ducts and vessels during LC; however, only fluorescent cholangiography (FC) offers a real-time visualization, however, it can be cost-prohibitive and requires specialized equipment and training. We conducted this study to describe the feasibility of an alternative technique to visualize the structures of Calot’s triangle in real-time, guiding the dissection to achieve a successful critical view of safety (CVS).

## Materials and methods

We conducted a single-center retrospective analysis of our prospectively constructed database, evaluating perioperative results from 10 non-consecutive patients in whom LC was performed using transillumination of Calot’s triangle (group A) by two experienced surgeons, with 50 patients (group B) who received conventional LC. Patients were matched for age, sex, and body mass index (BMI). Demographic data, type of surgery, operative time, bleeding, intraoperative and postoperative complications, and hospital stay were analyzed. When data were not available, we reviewed the patients’ medical records.

Data are presented as mean and standard deviation for continuous variables, and categorical variables are expressed in percentages. Student’s t-test and chi-square tests were employed to evaluate group differences. We considered a p-value lower than 0.05 as statistically significant. For statistical analyses, we used IBM SPSS Statistics for Windows, Version 24.0. (IBM Corp., Armonk, NY, USA).

Transillumination technique

We used an open (Hasson) technique to enter the abdominal cavity with a transverse supraumbilical incision of 10 to 12 mm, then CO_2_ pneumoperitoneum was initiated at 12 mmHg, a 10-mm/30° scope was introduced, and three additional ports were placed under direct visualization: a 10-mm subxiphoid port and two 5-mm subcostal ports.

**Figure 1:**
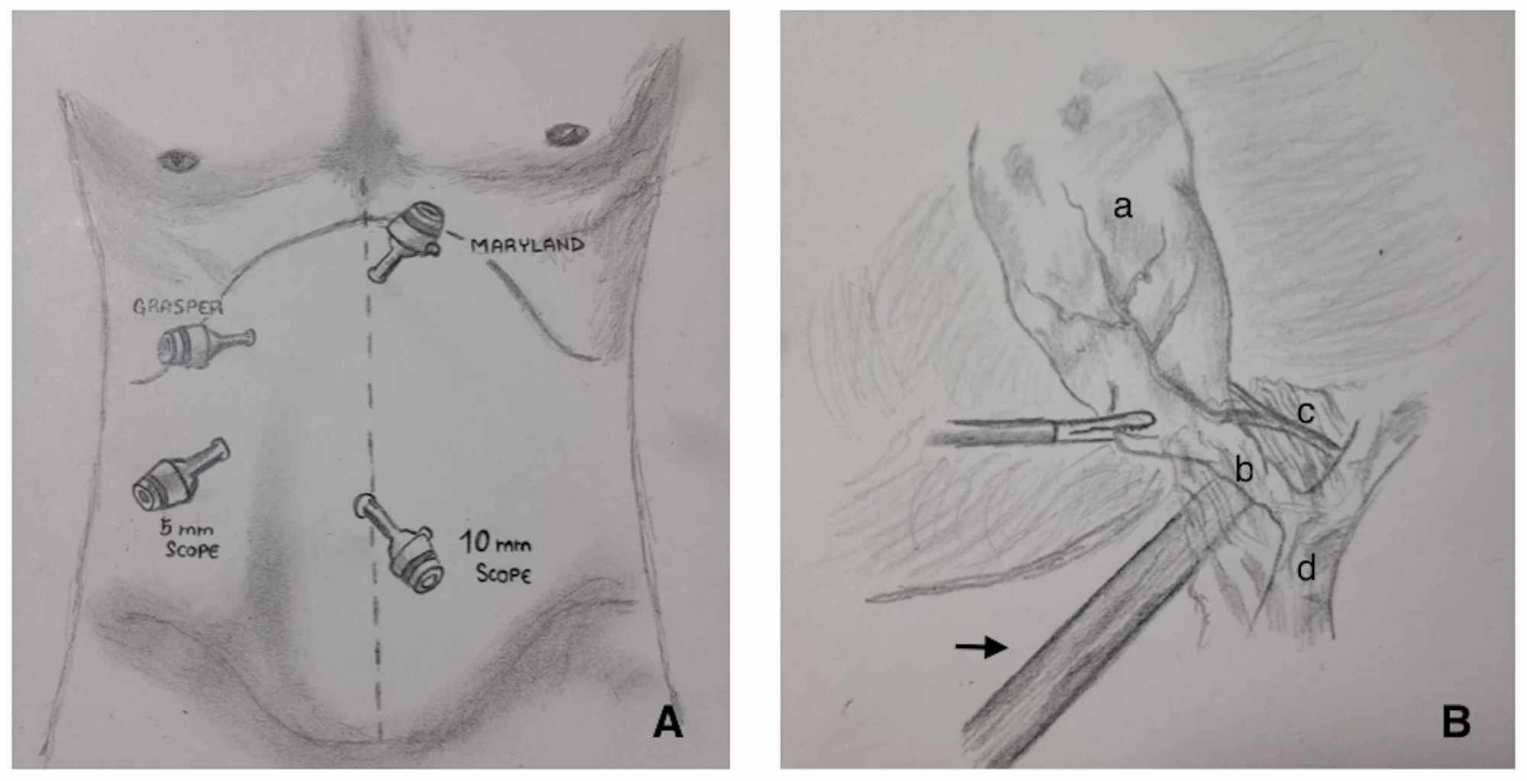
Illustration demonstrating our technique. (A) Port placement. (B) Placement of the second scope and its relation with adjacent structures (arrow: 5-mm scope; a: gallbladder; b: cystic duct; c: cystic artery; d: common bile duct).

**Figure 2:**
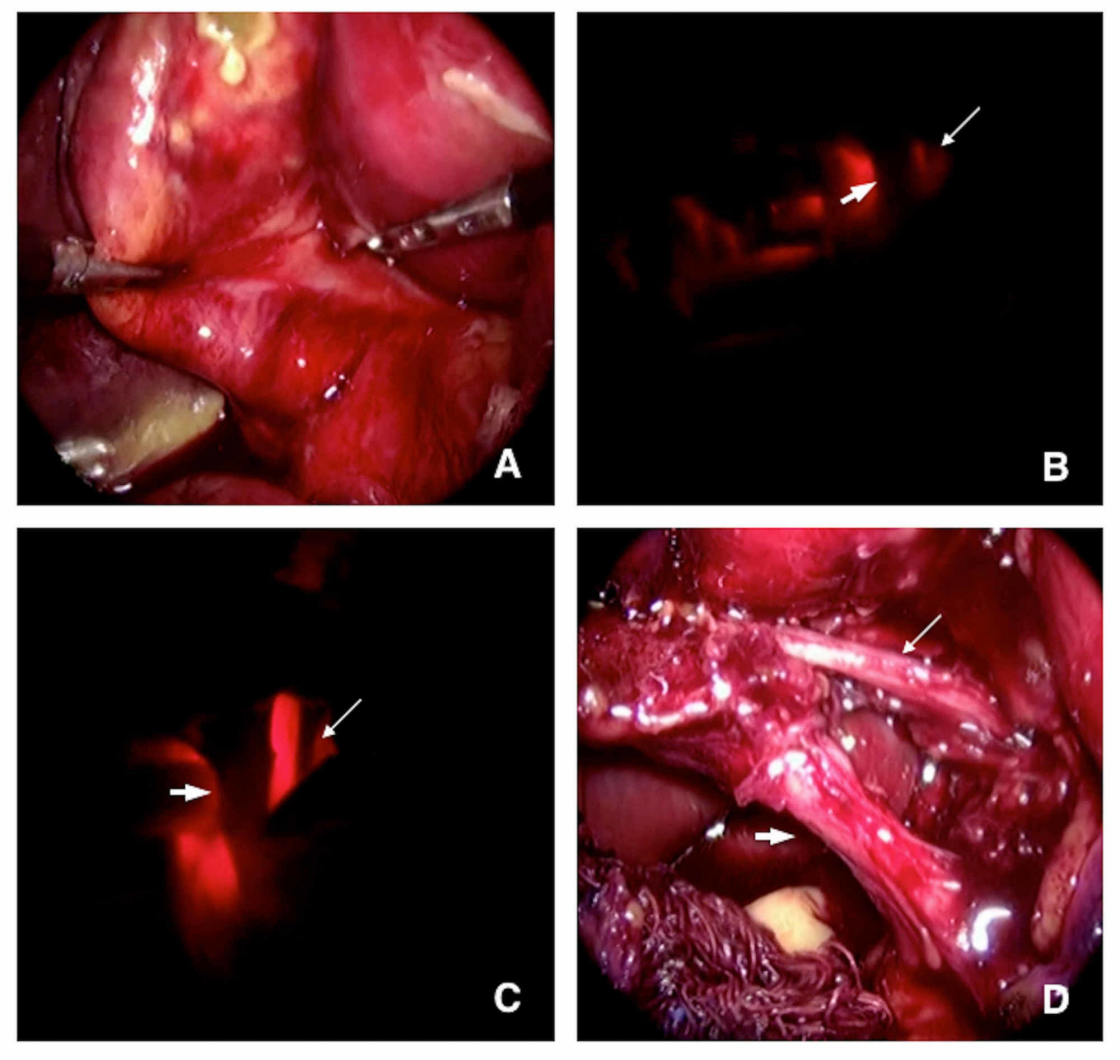
Transillumination on acute cholecystitis. (A) Initial view showing acute inflammation, no structure’s visualization. (B) Initial transillumination. (C) Transillumination after removal of the peritoneal layer. (D) View after dissection (thin arrow: cystic artery; thick arrow: cystic duct).

**Figure 3:**
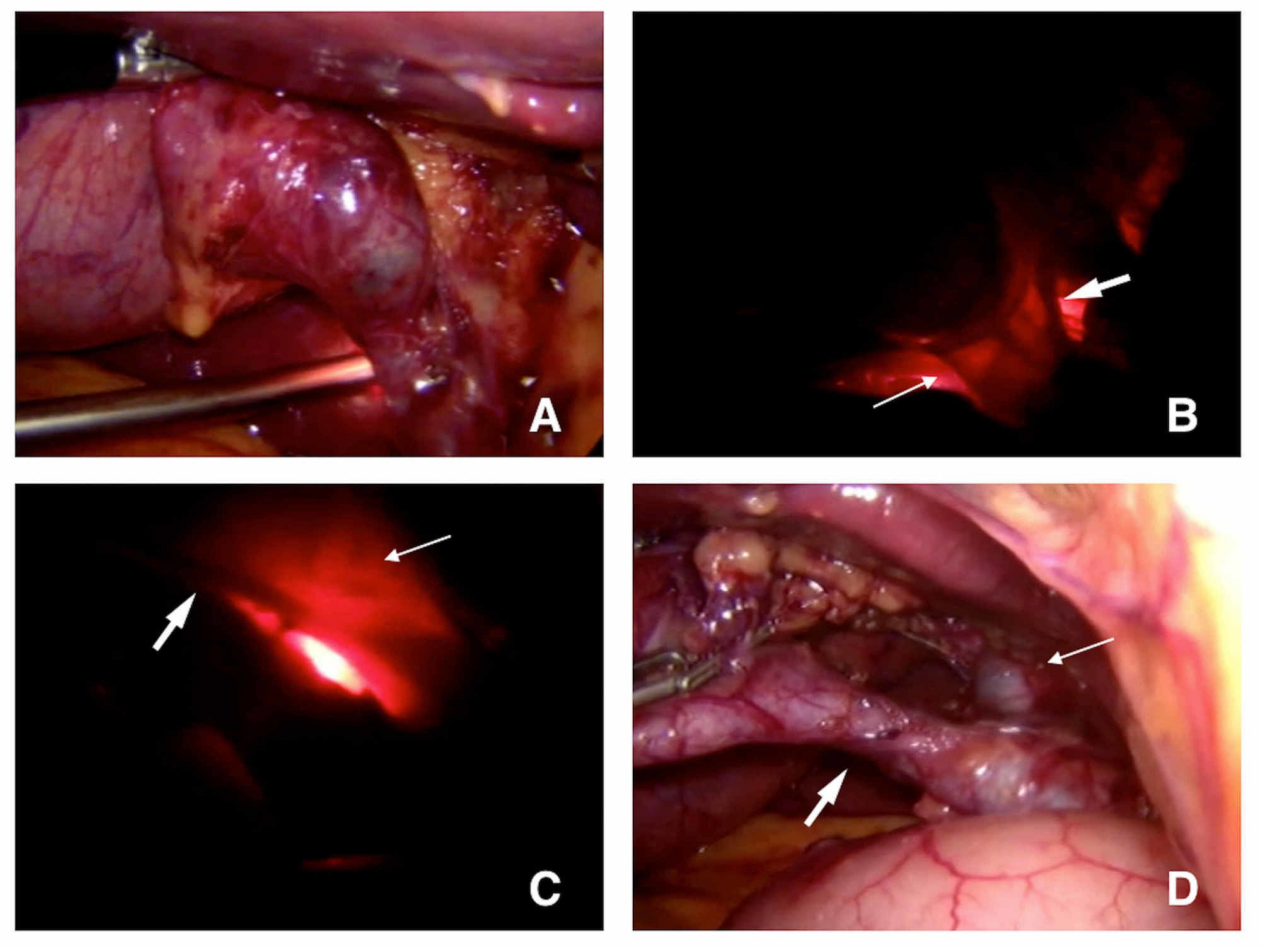
Transillumination on elective cholecystectomy. (A) Initial view and positioning of the second scope. (B) Initial transillumination (thin arrow: cystic duct; thick arrow: cystic artery). (C) Transillumination after initial dissection (thin arrow: structure free area; thick arrow: cystic artery). (D) View after dissection (thin arrow: common hepatic duct; thick arrow: cystic duct).

**Figure 4:**
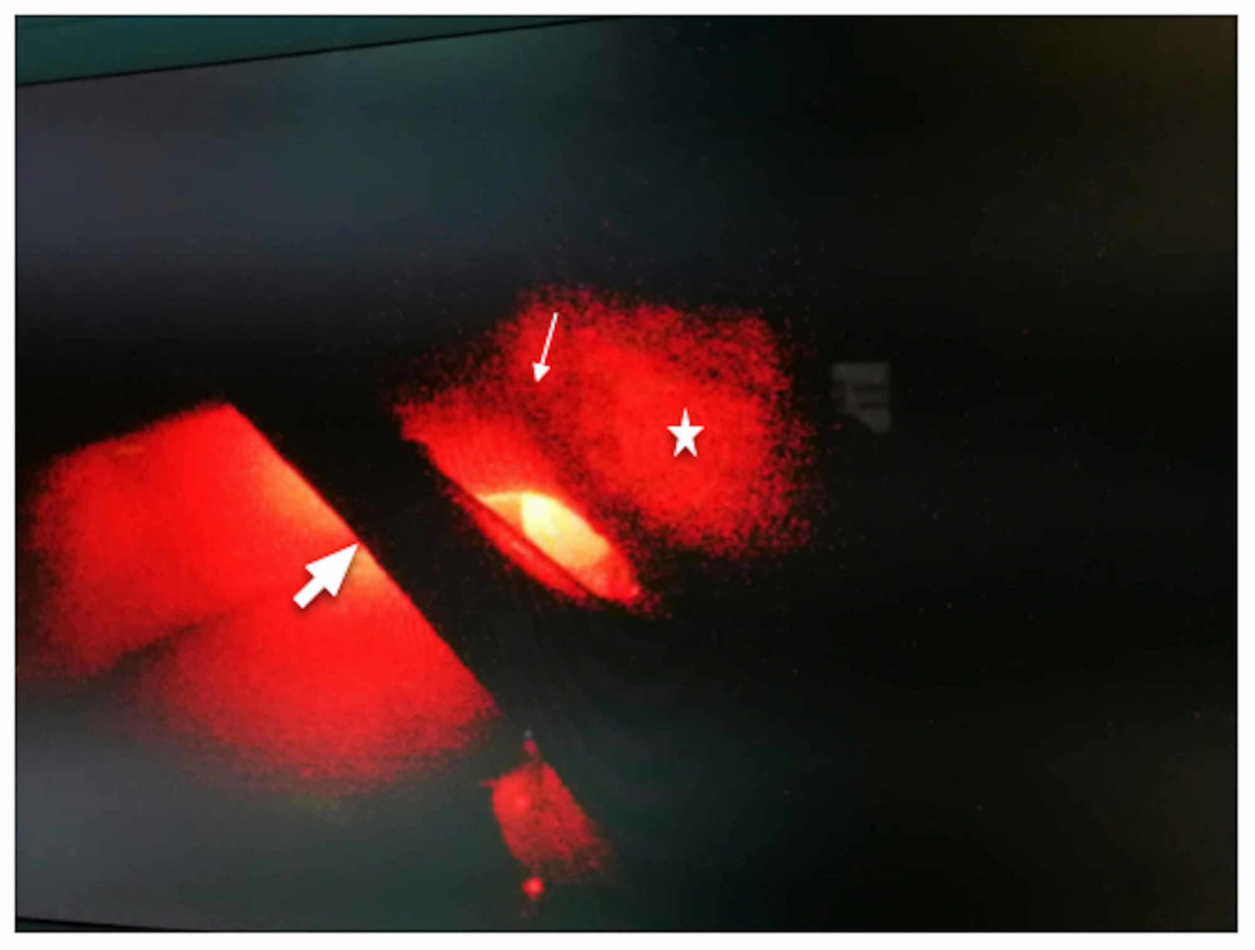
Transillumination after removal of the peritoneal layer. Thin arrow: cystic artery; thick arrow: cystic duct; star: structure free tissue.

After an initial laparoscopy, the camera directly faced the gallbladder, and early exposure was achieved using a 5-mm grasper to retract the infundibulum in a caudolateral direction. After that, a 5-mm/30° scope was introduced through the most lateral 5-mm port, placing it behind Calot’s triangle. To achieve transillumination of the area, the light of the 5-mm scope had to be rotated to face the triangle, and the 10-mm scope’s light has to be gradually decreased until visualization of the aimed structures is achieved (Figure 1). Depending on the anatomy of the area, the cystic duct (CD) and cystic artery (CA) may be identified. Another elective maneuver to clarify the anatomy in an attempt to identify the common bile duct (CBD) was performed by carefully advancing the 5-mm scope to locate the CBD’s 3 o’clock and 9 o’clock arteries. Once this procedure is concluded, the 10-mm scope’s light returns to optimal intensity, and the 5-mm scope is removed from the abdominal cavity to continue dissection (Figures 2, 3). However, when the visualization of the structures cannot be achieved, the posterior peritoneal layer could be opened followed by another attempt of transillumination (Figure 4). Dissection during transillumination is not advisable because the visualization of adjacent structures is reduced.

## Results

Group A consisted of nine women and one man, with a mean age of 50.7 (± 17.4) years and a mean BMI of 26.8 (±0.65) kg/m^2^. Three of the LCs in group A were emergency procedures (30%), and seven were elective LCs. All procedures for group A were performed for benign pathology. Group B had 40 women and 10 men, with a mean age of 49.7 (±15.2) years and a mean BMI of 27.5 (±4.5) kg/m^2^. Two of the group B cholecystectomies were emergency procedures (4%). There was no statistical difference in sex, age, or BMI between group A and group B (p=0.85, p=0.45, and p=0.65, respectively). The difference in the incidence of emergency versus elective procedure was significant (30% in group A vs. g4% in group B; p<0.01; Table 1).

**Table 1:** Comparison of demographic characteristics between group A (transillumination laparoscopic cholecystectomy) and group B (conventional laparoscopic cholecystectomy). Data are expressed as mean ± SD or as percentage (%). F, female; M, male; BMI, body mass index; SD, standard deviation

	Group A (n=10)	Group B (n=50)	p-Value
Mean age (years)	50.7 (±17.4)	49.7 (±15.2)	0.85
Sex (F/M)	9/1	40/10	0.45
BMI (kg/m^2^)	26.8 (±3.9)	27.5 (±4.5)	0.65
Emergency surgery	3 (30%)	2 (4%)	<0.01

Surgical outcomes for group A included a mean operative time of 90.1 (±29) minutes, bleeding of 28.5 (±17.4) mL, one complication (10%), and a mean hospital stay of 1.5 (±0.7) days. Group B had a mean operative time of 82.8 (±27) minutes, bleeding of 33.2 (±32.5) mL, four complications (8%), and a mean hospital stay of 1.58 (±1.2) days. There was no statistical difference between the groups regarding operative time (p=0.44), bleeding (p=0.66), number of complications (p=0.83), and mean hospital stay (p=0.85; Table 2).

**Table 2:** Comparison of surgical outcomes in group A (transillumination laparoscopic cholecystectomy) and group B (conventional laparoscopic cholecystectomy). Data expressed as mean ± SD or as percentage (%).

	Group A (n=10)	Group B (n=50)	p-Value
Time (minutes)	90.1 (±29)	82.8 (±27)	0.44
Bleeding (ml)	28.5 (±17.4)	33.2 (±32.5)	0.66
Complications	1 (10%)	4 (8%)	0.83
Mean length of stay (days)	1.5 (±0.7)	1.58 (±1.2)	0.85

The one complication noted in group A was Clavien-Dindo grade I. Specifically, it was a contained abdominal wall hematoma secondary to local anesthetic infiltration for the subxiphoid port, with no need for additional intervention. Of the four complications noted in group B, three were Clavien-Dindo grade I, including a Strasberg A BDI, treated conservatively, a hepatic subcapsular hematoma with no need of further interventions, and one liver bed bleeding with no need for transfusions or interventions. The other patient had a Clavien-Dindo grade III, developing a biloma that needed percutaneous management.

Group A had less bleeding than group B (28.5 ± 17.4 mL vs. 33.2 ± 32.5 mL) and longer operative time (90.1 ± 29 minutes vs. 82.9 ± 27 minutes). The difference was not statistically significant.

Visualization of structures was accomplished in all the procedures in group A. For the seven elective procedures, initial visualization of structures was achieved in two cases; visualization was achieved in the other five cases after the removal of the peritoneal layer. For the three emergency procedures, initial dissection was needed to achieve structure visualization with transillumination (Table 3).

**Table 3:** Visualization of Calot’s triangle structures with the use of transillumination initially and after peritoneal layer removal in both elective and emergency cholecystectomy. Data are expressed in number and percentage (%).

	Initial	After dissection
Elective	2 (28.6%)	5 (71.4%)
Emergency	0 (0%)	3 (100%)

## Discussion

The leading cause of a BDI was initially attributed to the learning curve and lack of technical skills, knowledge, or judgment in cholecystectomies. However, this cause accounts only for 3% of the cases [3]. The principal cause of BDI (97%) is the misinterpretation of biliary anatomy [3]. Injuries often arise from the misidentification of the CBD for the CD or by dissecting too close to the CBD or common hepatic duct (CHD). In such cases, the biliary anatomy was not fully recognized given the presence of connective tissue or inflammation; therefore, the wrong duct is separated, and the surgeon proceeds to remove the gallbladder from the liver. Any duct identified on this final step is often reported as an accessory structure, and the right hepatic artery (RHA) ends up being mistaken for the CA [3,4].

In 1995, Strasberg et al. proposed the CVS for conclusive anatomical identification to prevent BDI. CVS has since been validated worldwide and endorsed by many surgical societies as a feasible approach to a safe cholecystectomy [5,6]. Since the introduction of the CVS, many studies have validated its feasibility and utility in diminishing BDI, with some series even reporting zero incidences of BDI [7]. Eighty percent of BDIs occur while attempting a CVS [8], and Strasberg et al. published a three-step roadmap for avoiding BDI that consists of the following sequence [9]. First, surgeons should try to get a secure, anatomical identification of key structures. When this is not possible, the surgeon should not attempt a total cholecystectomy (“inflection point”). For finishing the operation safely, Strasberg’s last point includes conversion or bail out procedures such as cholecystostomy, subtotal cholecystectomy, either fenestrated or reconstituting, conversion, or even concluding the procedure and referring the patient appropriately [9].

Mascagni et al. emphasized a maneuver described by Strasberg et al. to achieve a complete CVS when cystic structures are short or the CA enters high into the gallbladder [10]. The maneuver consists of identifying the CA and dividing it to achieve a better exposition of the CD and, subsequently, the correct CVS. However, misidentification of the structures could lead to RHA injury or the CD itself, and, in fewer cases, a BDI could present [10]. Therefore, knowledge of the arterial and biliary anatomical variations is needed, and physicians should consider that in only 81.5% of cases the CA is found in the hepatocystic triangle [11,12].

Fluorescent cholangiography

Many studies have reported good results with the use of FC [4,13-16]. This method requires administration of indocyanine green (ICG), commonly 2.5 mg or 0.05 mg/kg, 30 minutes before surgery [15]. When near-infrared light is applied to the bile ducts, ICG generates fluorescence, and bile structures can be differentiated from surrounding tissue. An angiography may be performed by administrating a second bolus of ICG during surgery. After 30 seconds to two minutes, the CA might be observed by fluorescence. However, the procedure requires specialized equipment consisting of a small control unit, a charge-coupled device camera, a xenon light source, and a 10-mm laparoscope with specially coated lenses capable of transmitting near-infrared light [4].

The detection of the biliary system with FC differs among structures. In one study, CD was identified in 71.4% to 100% of the cases, CHD was identified in 33.3% to 100%, and the CBD was identified in 50% to 100% [4]. For studies comparing results before and after dissection, the sensitivity increases posterior to dissection for the CD from 72% to 95.1% [14]. In another study, the CA was identified in 85.9% of cases when a second bolus of ICG was administrated [15]. However, two conditions might diminish FC sensitivity: obesity and acute cholecystitis [4,15,16].

When compared with intraoperative cholangiography (IOC), there is moderate-quality evidence that visualization of the CD and CBD improves with FC (relative risk [RR]: 1.16 and 1.00, respectively), and low-quality evidence for CHD visualization (RR: 0.76) [15]. Furthermore, routine use of IOC for LC is debatable due to IOC’s limited availability and the requirements of staff trained on the equipment operation. Also, prior dissection and incision of the CD are required. Finally, both the patient and staff members are exposed to radiation [15].

To our knowledge, there are no previous records in the English literature of laparoscopic transillumination; the only precedent in the literature is from 2005 [17], and in 2008 [18], Nychytaĭlo et al. performed verification of biliary ducts and vessels by transillumination on LC and open cholecystectomy with a patented device with promising results. However, no images were provided to clarify the technique applied by the authors, and this method is rarely used [17,18].

In our study, the CA and CD were identified in 10 consecutive LCs by transillumination. In the majority of patients (8/10), the peritoneum covering Calot’s triangle needed to be opened prior to successful visualization. There were no biliary injuries in the transilluminated group compared with two in the conventional cholecystectomy group (2/50).

The fact that group A had significantly more emergency procedures might explain the mean longer operative time. Also, despite the higher proportion of emergency procedures in group A, the lower bleeding might indicate a better bleeding control due to the direct visualization of the vascular structures by transillumination and the recognition of a loose safe dissection area.

The main limitations of our study were the small sample size for group A and the retrospective, single-center design. There was also a level of inter-surgeon variability. Future studies could mitigate these limitations by using a prospective case-control study with larger sample sizes and standardizations to reduce inter-surgeon variability.

## Conclusions

Calot’s triangle transillumination is a promising approach for achieving a CVS during LC and represents an additional option for surgeons to visualize Calot’s triangle structures in order to improve dissection safety. This procedure helps to guide Calot’s triangle dissection as it highlights loose areas where safe dissection could be achieved; however, it does not replace the general safety principles for LC. The potential benefits of our transillumination method are its reproducibility and feasibility, and it could be attempted with alternative devices rather than a second scope as a light source. More studies on different centers are needed to optimize the procedure and validate this technique.
